# 1-*O*-Benzyl-2,3-*O*-iso­propyl­idene-6-*O*-tosyl-α-l-sorbo­furan­ose

**DOI:** 10.1107/S1600536813015638

**Published:** 2013-06-12

**Authors:** John H. Reed, Peter Turner, Atsushi Kato, Todd A. Houston, Michela I. Simone

**Affiliations:** a School of Chemistry (F11), University of Sydney, NSW 2006, Australia; b Crystal Structure Analysis Facility, School of Chemistry (F11), University of Sydney, NSW 2006, Australia; c Department of Hospital Pharmacy, University of Toyama, 2630, Sugitani, Toyama 930-0194, Japan; d Institute for Glycomics, Gold Coast Campus, Griffith University, Queensland 4222, Australia

## Abstract

In the title compound (systematic name: {(3a*S*,5*S*,6*R*,6a*S*)-3a-[(benz­yloxy)meth­yl]-6-hy­droxy-2,2-di­methyl­tetra­hydro­furo[2,3-*d*][1,3]dioxol-5-yl}methyl 4-methyl­benzene­sulfonate), C_23_H_28_O_8_S, the absolute structure and relative stereochemistry of the four chiral centres have been established by X-ray crystallography, with the absolute configuration inferred from the use of l-sorbose as the starting material. The central furan­ose ring adopts a slightly twisted envelope conformation (with the C atom bearing the methyl­benzene­sulfonate substituent as the flap) from which three substituents depart pseudo-axially (–CH_2_—*O*—benzyl, –OH and one acetonide O atom) and two substituents pseudo-equatorially (–CH_2_—*O*—tosyl and second acetonide O atom). The dioxalane ring is in a flattened envelope conformation with the fused CH C atom as the flap. In the crystal, mol­ecules pack in columns along [010] linked by O—H⋯O hydrogen bonds involving the furan­ose hy­droxy group and furan­ose ether O atom.

## Related literature

The title compound is a novel inter­mediate in the synthesis of 1-de­oxy­nojirimycin (DNJ) analogues. For examples of the use of monosaccharide starting materials in imino­sugar syntheses, see: Compain & Martin (2001 ▶); Cipolla *et al.* (2003 ▶); Best, Wang *et al.* (2010 ▶); Wilkinson *et al.* (2010 ▶); Nash *et al.* (2011 ▶); Zhang *et al.* (2011 ▶); Lenagh-Snow *et al.* (2011 ▶); Simone *et al.* (2012 ▶); Soengas *et al.* (2012 ▶); Kato *et al.* (2012 ▶). For examples of the synthesis of other biologically active compounds from monosaccharides, see: Compain *et al.* (2009 ▶); Sridhar *et al.* (2012 ▶); Das *et al.* (2012 ▶); Dhavale & Matin (2005 ▶); Compain & Martin (2001 ▶); Derosa & Maffioli (2012 ▶); Lew *et al.* (2000 ▶); Itzstein *et al.* (1993 ▶). For glycosidase inhibitors, see: Houston & Blanchfield (2003 ▶). For imino­sugars as glycosidase inhibitors, see: Zechel *et al.* (2003 ▶); de Melo *et al.* (2006 ▶); Compain & Martin (2007 ▶). For examples of the clinical uses of imino­sugars, see: Cox *et al.* (2003 ▶); Venier *et al.* (2012 ▶); Derosa & Maffioli (2012 ▶). For imino­sugars in the treatment of cancer, cystic fibrosis and viral diseases, see: Nishimura (2003 ▶); Lawton & Witty (2011 ▶); Best, Jenkinson *et al.* (2010 ▶); Compain & Martin (2007 ▶); Pollock *et al.* (2008 ▶). For the syntheses of DNJ and its analogues from l-sorbose, see: Beaupere *et al.* (1989 ▶); Masson *et al.* (2000 ▶); Tamayo *et al.* (2010 ▶); O’Brien & Murphy (2011 ▶). For the synthesis of 1-*O*-benzoyl-2,3-*O*-iso­propyl­idene-6-*O*-tosyl-α-l-sorbo­furan­ose, which bears structural similarity to the title compound, see: Fehér & Vargha (1966 ▶).
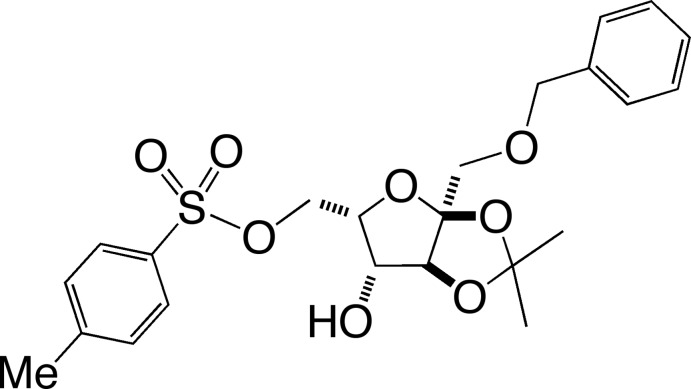


## Experimental

### 

#### Crystal data

C_23_H_28_O_8_S
*M* *_r_* = 464.51
Monoclinic, 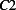

*a* = 22.6192 (3) Å
*b* = 5.5649 (1) Å
*c* = 19.0631 (3) Å
β = 104.696 (2)°
*V* = 2321.04 (6) Å^3^
*Z* = 4
Cu *K*α radiation
μ = 1.64 mm^−1^
*T* = 150 K
0.29 × 0.06 × 0.02 mm

#### Data collection

Agilent SuperNova (Dual, Cu at zero, Atlas) diffractometer
Absorption correction: multi-scan (*CrysAlis PRO*; Agilent, 2011 ▶) *T*_min_ = 0.628, *T*_max_ = 1.000
24544 measured reflections
4672 independent reflections
4541 reflections with *I* > 2σ(*I*)
*R*_int_ = 0.031

#### Refinement

*R*[*F* ^2^ > 2σ(*F* ^2^)] = 0.044
*wR*(*F* ^2^) = 0.127
*S* = 1.16
4672 reflections
293 parameters
1 restraint
H-atom parameters constrained
Δρ_max_ = 0.48 e Å^−3^
Δρ_min_ = −0.27 e Å^−3^
Absolute structure: Flack (1983 ▶), 2165 Friedel pairs
Flack parameter: 0.000 (15)

### 

Data collection: *CrysAlis PRO* (Agilent, 2011 ▶); cell refinement: *CrysAlis PRO*; data reduction: *CrysAlis PRO*; program(s) used to solve structure: *SIR97* (Altomare *et al.*, 1999 ▶); program(s) used to refine structure: *SHELXL97* (Sheldrick, 2008 ▶); molecular graphics: *Xtal3*.6 (Hall *et al.*, 1999 ▶), *ORTEPII* (Johnson, 1976 ▶), *SHELXLE* (Hübschle *et al.*, 2011 ▶), *Mercury* (Macrae *et al.*, 2006 ▶) and *WinGX* (Farrugia, 2012 ▶); software used to prepare material for publication: *publCIF* (Westrip, (2010 ▶).

## Supplementary Material

Crystal structure: contains datablock(s) I, global. DOI: 10.1107/S1600536813015638/lh5615sup1.cif

Structure factors: contains datablock(s) I. DOI: 10.1107/S1600536813015638/lh5615Isup2.hkl

Supplementary material file. DOI: 10.1107/S1600536813015638/lh5615Isup3.cml

Additional supplementary materials:  crystallographic information; 3D view; checkCIF report

## Figures and Tables

**Table 1:** Hydrogen-bond geometry (Å, °)

*D*—H⋯*A*	*D*—H	H⋯*A*	*D*⋯*A*	*D*—H⋯*A*
O3—H3*O*⋯O4^i^	0.84	1.98	2.812 (2)	174

Symmetry code: (i) 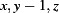.

**Fig. 1.**
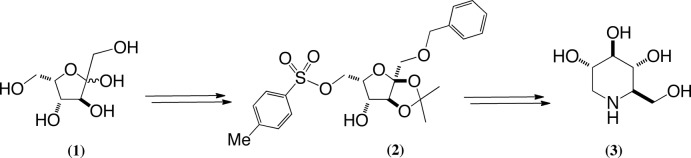
Relationship of L-sorbose (1) starting material to title compound (2) and deoxynojirimycin (DNJ) (3)

**Fig. 2.**
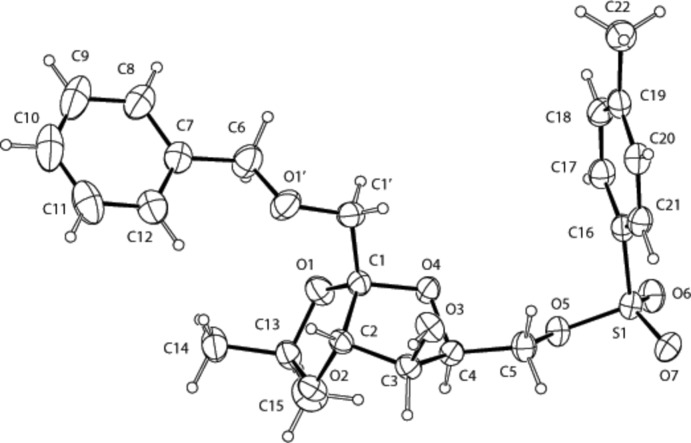
The molecular structure of the title compound, with anisotropic displacement ellipsoids shown at the 50% probability level.

**Fig. 3.**
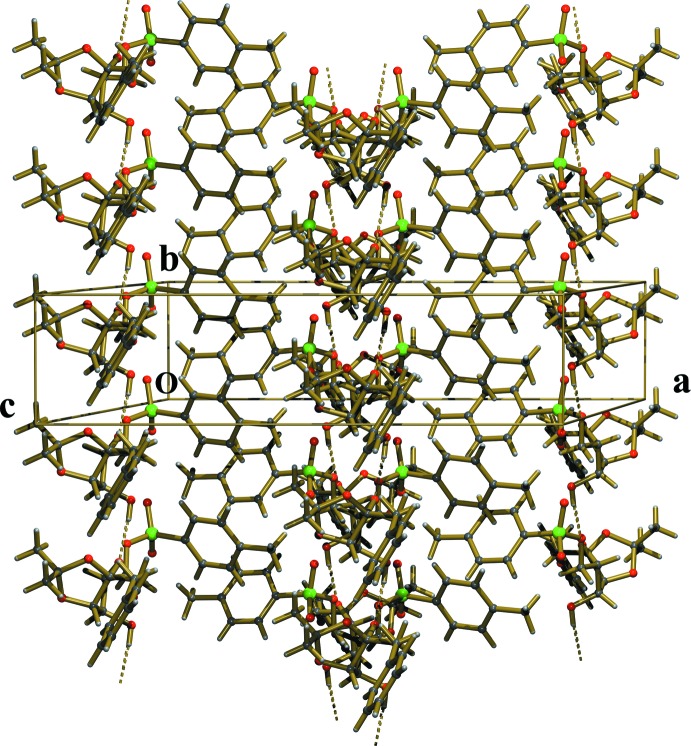
Molecular packing diagram (Macrae et al., 2006) with respect to the unit cell. The molecules stack in columns parallel to the b axis, with molecules within a column linked by intermolecular hydrogen bonds (dashed lines) between the hydroxy O3 moiety and the furanose oxygen O4.

## supplementary crystallographic information

### Comment

Monosaccharides provide a vast and formidable chiral pool of starting materials, whose utilization continues to expand in the enantiospecific syntheses of natural products (Sridhar *et al.*, 2012; Das *et al.*, 2012), in particular of carbohydrate mimetics of the carbasugar (Derosa & Maffioli, 2012; Lew *et al.* 2000), C-glycoside (Compain & Martin, 2001; Dhavale & Matin, 2005; Compain *et al.*, 2009), THP (Itzstein *et al.* 1993) and iminosugar (Cipolla *et al.*, 2003; Wilkinson *et al.* (2010); Nash *et al.*, 2011; Zhang *et al.*, 2011; Lenagh-Snow *et al.*, 2011; Simone *et al.*, 2012; Soengas *et al.* 2012; Best, Wang *et al.* 2010; Kato *et al.*, 2012) types, to mention a few.

Iminosugars have been recognized as a class of potent inhibitors of glycosidase enzymes (Houston & Blanchfield 2003; Zechel *et al.*, 2003; de Melo *et al.*, 2006; Compain and Martin, 2007). These potent biological activities have culminated in the marketing of *N*-butyl-DNJ for the treatment of Gauchers disease (Miglustat) (Cox *et al.*, 2003; Venier *et al.*, 2012), *N*-hydroxyethyl-DNJ for type II diabetes (Miglitol) (Derosa & Maffioli, 2012) while other iminosugars have opened up new in-roads in the treatment of cancer (Nishimura, 2003; Lawton & Witty 2011), cystic fibrosis (Best, Jenkinson *et al.*, 2010) and as antivirals (Compain & Martin, 2007; Pollock *et al.* 2008).

The first synthesis of DNJ (3 in Fig. 1) from starting material L-sorbose (1) utilized triphenylphosphine, carbon tetrabromide and lithium azide to effect the key transformation which installs an azido group in place of the C5 hydroxy (Beaupere *et al.*, 1989). Syntheses of further DNJ derivatives from L-sorbose have been reported (Masson *et al.*, 2000; Tamayo *et al.*, 2010; O'Brien & Murphy, 2011). The title compound (2 in Fig. 1) bears orthogonal protecting groups on four of the five hydroxy groups, thus opening up points of synthetic divergence to novel classes of iminosugar glycomimetics based on a DNJ scaffold.

In the title compound (Fig. 2), the central furanose ring adopts a slightly twisted envelope conformation with C4 forming the flap. The O1 and C5 substituents are positioned pseudo-equatorially, while the C1', O2 and O3 substituents are positioned pseudo-axially. The dioxalane ring is in a flattened envelope conformation with C2 forming the flap. The title compound bears structural similarity to 1-*O*-benzoyl-2,3-*O*-isopropylidene-6-*O*-tosyl-α-L-sorbofuranose ([α]D20 0° (c = 1 in CHCl_3_); m.p. 428–429 K (decomposition)) (Fehér & Vargha, 1966). In the crystal, molecules pack in columns in the [010] direction linked by O—H···O hydrogen bonds invovling the furanose hydroxy group and furanose ether oxygen atom (Fig. 3).

### Experimental

Triethylamine (3.43 ml, 24.6 mmol), 4-dimethylaminopyridine (120 mg, 982 µmol), and 4-toluenesulfonyl chloride (2.35 g, 12.3 mmol) were carefully added in succession to a stirring solution of 1-*O*-benzyl-2,3-*O*-isopropylidene-α-L-sorbofuranose (3.06 g, 9.84 mmol) in dichloromethane (200 ml) under an inert atmosphere. The reaction mixture was stirred for 28 h at room temperature, after which, analysis by TLC (acetone/hexane 1:2) showed total consumption of the starting material (Rf = 0.18) and formation of a UV-active product (Rf = 0.44). The crude mixture was washed with an aqueous hydrochloric acid solution (1 *M*, 100 ml) and the organic layers collected, dried over magnesium sulfate, filtered and concentrated *in vacuo*. The compound crystallized on standing as a pale yellow solid in quantitative yield (4.57 g).

### Refinement

All H atoms attached to C atoms were positioned geometrically, and allowed to ride on their parent atoms, with C—H bond lengths of 0.95 Å (Ar—H), 1.0 (CH), 0.99 Å (CH2) or 0.98 Å (CH3), and isotropic displacement parameters set to 1.2 (CH and CH2) or 1.5 times (CH3) the *U*_eq_ of the parent atom. The H atom attached to O3 was positioned geometrically and allowed to ride on the parent atom, with an O—H bond length of 0.84 Å and isotropic displacement parameters set to 1.5*U*_eq_(O).

### Crystal data

**Table:**

C_23_H_28_O_8_S	*F*(000) = 984
*M**_r_* = 464.51	*D*_x_ = 1.329 Mg m^−^^3^
Monoclinic, *C*2	Cu *K*α radiation, λ = 1.5418 Å
Hall symbol: C 2y	Cell parameters from 13737 reflections
*a* = 22.6192 (3) Å	θ = 4.0–76.2°
*b* = 5.5649 (1) Å	µ = 1.64 mm^−^^1^
*c* = 19.0631 (3) Å	*T* = 150 K
β = 104.696 (2)°	Blade, colourless
*V* = 2321.04 (6) Å^3^	0.29 × 0.06 × 0.02 mm
*Z* = 4	

### Data collection

**Table:**

Agilent SuperNova (Dual, Cu at zero, Atlas) diffractometer	4672 independent reflections
Radiation source: SuperNova (Cu) X-ray Source	4541 reflections with *I* > 2σ(*I*)
Mirror monochromator	*R*_int_ = 0.031
Detector resolution: 10.5861 pixels mm^-1^	θ_max_ = 76.4°, θ_min_ = 4.0°
ω scans	*h* = −28→28
Absorption correction: multi-scan (*CrysAlis PRO*; Agilent, 2011)	*k* = −7→6
*T*_min_ = 0.628, *T*_max_ = 1.000	*l* = −24→24
24544 measured reflections	

### Refinement

**Table:**

Refinement on *F*^2^	Secondary atom site location: difference Fourier map
Least-squares matrix: full	Hydrogen site location: inferred from neighbouring sites
*R*[*F*^2^ > 2σ(*F*^2^)] = 0.044	H-atom parameters constrained
*wR*(*F*^2^) = 0.127	*w* = 1/[σ^2^(*F*_o_^2^) + (0.1*P*)^2^] where *P* = (*F*_o_^2^ + 2*F*_c_^2^)/3
*S* = 1.16	(Δ/σ)_max_ = 0.001
4672 reflections	Δρ_max_ = 0.48 e Å^−^^3^
293 parameters	Δρ_min_ = −0.27 e Å^−^^3^
1 restraint	Absolute structure: Flack (1983), 2165 Friedel pairs
Primary atom site location: structure-invariant direct methods	Flack parameter: 0.000 (15)

### Special details

**Table:**

Experimental. Analysis: [α]_D_^26^ 0.20° (*c* 0.2 in CHCl_3_); IR (KBr, cm^-1^): 3594-3205 (*br*, OH), 3095, 3046 (w, ArC-H), 2984, 2950, 2925, 2864 (st, alkyl C-H), 1369, 1178 (*s*, S=O); ^1^H NMR (CDCl_3_, 400 MHz, p.p.m.): 1.27, 1.46 [2 x 3H, 2 x *s*, C(CH_3_)_2_], 2.43 [3H, *s*, TsCH_3_], 3.49 [1H, *d*, J_H3,H4_ = 11.8 Hz, H3], 3.57 [1H, *d*, J_H1,H1'_ = 10.1 Hz, H1], 3.76 [1H, *d*, J_H1',H1_ = 10.0 Hz, H1'], 4.05 [1H, *dd*, J_H4,H3_ = 11.5 Hz, J_H4,H5_ = 2.5 Hz, H4], 4.16 [1H, *dd*, J_H6,H5_ = 6.9 Hz, J_H6,H6'_ = 10.6 Hz, H6], 4.30 [1H, *dd*, J_H6',H5_ = 4.9, J_H6',H6_ = 10.6 Hz, H6'], 4.36 [1H, *s*, OH], 4.40 [1H, *ddd*, J_H5,H4_ = 2.5 Hz, J_H5,H6'_ = 4.7 Hz, J_H5,H6_ = 7.2 Hz, H5], 4.52 [1H, *d*, J_CHA_*_HB_*_,C_*_HA_*_HB_ = 11.7 Hz, CH_A_*H_B_* (Bn)], 4.59 [1H, *d*, J_C_*_HA_*_HB,CHA_*_HB_* = 11.7 Hz, C*H_A_*H_B_ (Bn)], 7.22-7.26 [2H, *m*, 2 x ArHs (Bn-*o*)], 7.30-7.37 [5H, *m*, 2 x ArHs (Ts) and 3 x ArHs (Bn-*m*,*p*)], 7.81 [2H, d, ArHs (Ts)]; ^13^C NMR (CDCl_3_, 100 MHz, p.p.m.): 21.7 [CH_3_ (Ts)], 26.1, 27.2 [2 x CH_3_], 68.1 [C6], 71.3 [C1], 74.2 [CH_2_(Bn)], 74.4 [C4], 79.8 [C5], 86.4 [C3], 112.8 [C2], 112.9 [Cq acetonide], 128.0 [2 x ArCs (Bn-*o*)], 128.2 [2 x ArCs (Ts)], 128.5 [1 x ArC (Bn-*p*)], 128.8, 129.9 [2 x ArCs (Bn-*m*) and 2 x ArCs (Ts)], 133.0 [*Cq*-CH_3_], 136.5 [Cq (Bn)], 144.9 [Cq-S]; HRMS (ESI^+^): found 487.13977 [M+Na]^+^ C_23_H_28_NaO_8_S, requires 487.13971. M.p.: 377-378K
Geometry. All e.s.d.'s (except the e.s.d. in the dihedral angle between two l.s. planes) are estimated using the full covariance matrix. The cell e.s.d.'s are taken into account individually in the estimation of e.s.d.'s in distances, angles and torsion angles; correlations between e.s.d.'s in cell parameters are only used when they are defined by crystal symmetry. An approximate (isotropic) treatment of cell e.s.d.'s is used for estimating e.s.d.'s involving l.s. planes.
Refinement. Refinement of *F*^2^ against ALL reflections. The weighted *R*-factor *wR* and goodness of fit *S* are based on *F*^2^, conventional *R*-factors *R* are based on *F*, with *F* set to zero for negative *F*^2^. The threshold expression of *F*^2^ > σ(*F*^2^) is used only for calculating *R*-factors(gt) *etc*. and is not relevant to the choice of reflections for refinement. *R*-factors based on *F*^2^ are statistically about twice as large as those based on *F*, and *R*- factors based on ALL data will be even larger.

### Fractional atomic coordinates and isotropic or equivalent isotropic displacement parameters (Å^2^)

**Table:**

	*x*	*y*	*z*	*U*_iso_*/*U*_eq_	
S1	0.404262 (19)	0.50470 (8)	0.48560 (2)	0.02722 (14)	
O1	0.44717 (7)	0.4027 (3)	0.17231 (8)	0.0355 (3)	
O1'	0.34123 (8)	0.1296 (4)	0.09744 (8)	0.0438 (4)	
O2	0.50981 (6)	0.0816 (3)	0.21233 (8)	0.0313 (3)	
O3	0.39754 (6)	−0.1689 (3)	0.29897 (8)	0.0316 (3)	
H3O	0.4000	−0.3170	0.2916	0.047*	
O4	0.41584 (6)	0.3389 (2)	0.27843 (7)	0.0279 (3)	
O5	0.43901 (6)	0.4404 (3)	0.42627 (7)	0.0289 (3)	
O6	0.41714 (7)	0.7530 (3)	0.50128 (8)	0.0354 (3)	
O7	0.41841 (7)	0.3306 (3)	0.54278 (7)	0.0345 (3)	
C1	0.41496 (8)	0.2518 (4)	0.20750 (9)	0.0269 (4)	
C1'	0.34827 (9)	0.2402 (5)	0.16634 (11)	0.0383 (5)	
H1'A	0.3252	0.1477	0.1949	0.046*	
H1'B	0.3312	0.4048	0.1596	0.046*	
C2	0.45054 (8)	0.0140 (4)	0.21843 (9)	0.0259 (3)	
H2	0.4311	−0.1123	0.1826	0.031*	
C3	0.45313 (8)	−0.0559 (4)	0.29689 (10)	0.0270 (4)	
H3	0.4895	−0.1575	0.3191	0.032*	
C4	0.45641 (8)	0.1905 (3)	0.33235 (9)	0.0254 (4)	
H4	0.4991	0.2542	0.3426	0.030*	
C5	0.43459 (9)	0.1918 (4)	0.40058 (10)	0.0302 (4)	
H5A	0.3918	0.1348	0.3904	0.036*	
H5B	0.4605	0.0855	0.4376	0.036*	
C6	0.32399 (13)	0.2890 (5)	0.03842 (12)	0.0436 (5)	
H6A	0.3540	0.4216	0.0443	0.052*	
H6B	0.2835	0.3594	0.0370	0.052*	
C7	0.32113 (10)	0.1574 (5)	−0.03123 (11)	0.0365 (5)	
C8	0.28553 (11)	0.2509 (6)	−0.09630 (12)	0.0459 (6)	
H8	0.2630	0.3950	−0.0961	0.055*	
C9	0.28292 (15)	0.1343 (7)	−0.16111 (13)	0.0602 (9)	
H9	0.2589	0.1997	−0.2052	0.072*	
C10	0.31489 (15)	−0.0766 (7)	−0.16223 (15)	0.0611 (9)	
H10	0.3129	−0.1558	−0.2069	0.073*	
C11	0.34979 (14)	−0.1721 (6)	−0.09822 (17)	0.0563 (7)	
H11	0.3716	−0.3177	−0.0987	0.068*	
C12	0.35291 (11)	−0.0536 (5)	−0.03250 (13)	0.0428 (5)	
H12	0.3771	−0.1190	0.0115	0.051*	
C13	0.50589 (9)	0.3003 (4)	0.17266 (11)	0.0313 (4)	
C14	0.50694 (13)	0.2538 (6)	0.09465 (13)	0.0496 (6)	
H14A	0.4735	0.1446	0.0721	0.074*	
H14B	0.5461	0.1807	0.0934	0.074*	
H14C	0.5019	0.4060	0.0679	0.074*	
C15	0.55631 (12)	0.4642 (5)	0.21200 (15)	0.0481 (6)	
H15A	0.5959	0.3946	0.2112	0.072*	
H15B	0.5541	0.4834	0.2624	0.072*	
H15C	0.5518	0.6215	0.1881	0.072*	
C16	0.32657 (8)	0.4727 (4)	0.44051 (9)	0.0281 (4)	
C17	0.29721 (10)	0.6518 (4)	0.39353 (11)	0.0324 (4)	
H17	0.3195	0.7858	0.3828	0.039*	
C18	0.23503 (10)	0.6319 (5)	0.36260 (11)	0.0355 (4)	
H18	0.2147	0.7545	0.3308	0.043*	
C19	0.20172 (9)	0.4365 (4)	0.37695 (10)	0.0329 (4)	
C20	0.23236 (10)	0.2542 (4)	0.42171 (11)	0.0332 (4)	
H20	0.2105	0.1161	0.4302	0.040*	
C21	0.29487 (9)	0.2718 (4)	0.45432 (10)	0.0307 (4)	
H21	0.3154	0.1482	0.4855	0.037*	
C22	0.13348 (10)	0.4233 (6)	0.34609 (13)	0.0456 (6)	
H22A	0.1234	0.4671	0.2946	0.068*	
H22B	0.1133	0.5349	0.3723	0.068*	
H22C	0.1194	0.2593	0.3513	0.068*	

### Atomic displacement parameters (Å^2^)

**Table:**

	*U*^11^	*U*^22^	*U*^33^	*U*^12^	*U*^13^	*U*^23^
S1	0.0334 (2)	0.0263 (3)	0.0226 (2)	−0.00197 (16)	0.00836 (15)	−0.00147 (16)
O1	0.0404 (7)	0.0297 (8)	0.0412 (7)	0.0075 (6)	0.0190 (6)	0.0082 (6)
O1'	0.0522 (9)	0.0409 (10)	0.0313 (7)	0.0102 (7)	−0.0020 (6)	−0.0056 (7)
O2	0.0278 (6)	0.0284 (8)	0.0400 (7)	0.0039 (5)	0.0129 (5)	0.0037 (6)
O3	0.0356 (7)	0.0208 (7)	0.0417 (7)	−0.0025 (5)	0.0161 (6)	−0.0016 (5)
O4	0.0374 (7)	0.0213 (7)	0.0243 (6)	0.0056 (5)	0.0067 (5)	−0.0019 (5)
O5	0.0347 (6)	0.0267 (8)	0.0275 (6)	−0.0050 (5)	0.0121 (5)	−0.0029 (5)
O6	0.0441 (7)	0.0297 (8)	0.0337 (6)	−0.0060 (6)	0.0123 (6)	−0.0082 (6)
O7	0.0395 (7)	0.0384 (9)	0.0246 (6)	−0.0005 (6)	0.0061 (5)	0.0043 (6)
C1	0.0299 (8)	0.0266 (10)	0.0246 (8)	0.0038 (7)	0.0075 (7)	−0.0011 (7)
C1'	0.0326 (9)	0.0459 (13)	0.0328 (9)	0.0097 (9)	0.0017 (8)	−0.0080 (9)
C2	0.0265 (7)	0.0232 (9)	0.0287 (8)	0.0022 (7)	0.0083 (6)	−0.0027 (7)
C3	0.0288 (8)	0.0225 (10)	0.0305 (8)	0.0028 (7)	0.0090 (6)	0.0013 (7)
C4	0.0292 (8)	0.0217 (9)	0.0249 (8)	0.0005 (7)	0.0059 (6)	−0.0003 (7)
C5	0.0380 (9)	0.0256 (10)	0.0291 (8)	−0.0023 (7)	0.0125 (7)	−0.0020 (7)
C6	0.0591 (13)	0.0344 (13)	0.0342 (10)	0.0076 (11)	0.0061 (9)	0.0002 (9)
C7	0.0381 (10)	0.0383 (12)	0.0337 (10)	−0.0063 (9)	0.0102 (8)	−0.0017 (9)
C8	0.0475 (12)	0.0535 (16)	0.0346 (10)	−0.0093 (11)	0.0065 (9)	0.0032 (10)
C9	0.0682 (17)	0.081 (2)	0.0319 (11)	−0.0281 (17)	0.0130 (11)	−0.0017 (13)
C10	0.0691 (17)	0.079 (2)	0.0430 (12)	−0.0328 (17)	0.0275 (12)	−0.0210 (14)
C11	0.0620 (15)	0.0521 (16)	0.0651 (16)	−0.0171 (13)	0.0350 (13)	−0.0228 (14)
C12	0.0424 (11)	0.0429 (15)	0.0447 (11)	−0.0075 (10)	0.0143 (9)	−0.0080 (10)
C13	0.0350 (9)	0.0299 (11)	0.0327 (9)	0.0028 (8)	0.0153 (7)	0.0020 (8)
C14	0.0604 (14)	0.0579 (17)	0.0359 (11)	0.0145 (13)	0.0223 (10)	0.0010 (11)
C15	0.0476 (12)	0.0399 (15)	0.0595 (14)	−0.0129 (10)	0.0182 (10)	−0.0004 (11)
C16	0.0327 (8)	0.0286 (11)	0.0247 (7)	0.0014 (7)	0.0105 (6)	−0.0002 (7)
C17	0.0405 (10)	0.0279 (11)	0.0305 (8)	−0.0002 (8)	0.0122 (7)	0.0037 (8)
C18	0.0419 (10)	0.0355 (12)	0.0287 (9)	0.0067 (9)	0.0081 (7)	0.0033 (8)
C19	0.0347 (9)	0.0385 (12)	0.0273 (8)	0.0014 (8)	0.0113 (7)	−0.0059 (8)
C20	0.0391 (10)	0.0307 (11)	0.0319 (9)	−0.0055 (8)	0.0127 (7)	−0.0024 (8)
C21	0.0385 (9)	0.0284 (11)	0.0264 (8)	−0.0018 (8)	0.0104 (7)	0.0022 (7)
C22	0.0367 (10)	0.0585 (16)	0.0400 (11)	−0.0008 (10)	0.0069 (8)	−0.0096 (11)

### Geometric parameters (Å, º)

**Table:**

S1—O6	1.4278 (17)	C8—C9	1.384 (4)
S1—O7	1.4326 (16)	C8—H8	0.9500
S1—O5	1.5736 (13)	C9—C10	1.382 (6)
S1—C16	1.7585 (19)	C9—H9	0.9500
O1—C1	1.391 (2)	C10—C11	1.380 (5)
O1—C13	1.444 (2)	C10—H10	0.9500
O1'—C6	1.408 (3)	C11—C12	1.402 (4)
O1'—C1'	1.422 (3)	C11—H11	0.9500
O2—C13	1.424 (3)	C12—H12	0.9500
O2—C2	1.425 (2)	C13—C15	1.504 (3)
O3—C3	1.415 (2)	C13—C14	1.516 (3)
O3—H3O	0.8400	C14—H14A	0.9800
O4—C1	1.432 (2)	C14—H14B	0.9800
O4—C4	1.450 (2)	C14—H14C	0.9800
O5—C5	1.463 (2)	C15—H15A	0.9800
C1—C1'	1.515 (3)	C15—H15B	0.9800
C1—C2	1.535 (3)	C15—H15C	0.9800
C1'—H1'A	0.9900	C16—C21	1.389 (3)
C1'—H1'B	0.9900	C16—C17	1.391 (3)
C2—C3	1.532 (2)	C17—C18	1.385 (3)
C2—H2	1.0000	C17—H17	0.9500
C3—C4	1.522 (3)	C18—C19	1.389 (3)
C3—H3	1.0000	C18—H18	0.9500
C4—C5	1.504 (2)	C19—C20	1.392 (3)
C4—H4	1.0000	C19—C22	1.508 (3)
C5—H5A	0.9900	C20—C21	1.396 (3)
C5—H5B	0.9900	C20—H20	0.9500
C6—C7	1.503 (3)	C21—H21	0.9500
C6—H6A	0.9900	C22—H22A	0.9800
C6—H6B	0.9900	C22—H22B	0.9800
C7—C12	1.380 (4)	C22—H22C	0.9800
C7—C8	1.397 (3)		
			
O6—S1—O7	120.05 (10)	C9—C8—C7	120.2 (3)
O6—S1—O5	104.95 (8)	C9—C8—H8	119.9
O7—S1—O5	109.71 (9)	C7—C8—H8	119.9
O6—S1—C16	109.01 (10)	C10—C9—C8	120.6 (3)
O7—S1—C16	107.82 (9)	C10—C9—H9	119.7
O5—S1—C16	104.19 (8)	C8—C9—H9	119.7
C1—O1—C13	110.70 (15)	C11—C10—C9	119.8 (3)
C6—O1'—C1'	114.1 (2)	C11—C10—H10	120.1
C13—O2—C2	109.61 (14)	C9—C10—H10	120.1
C3—O3—H3O	109.5	C10—C11—C12	119.8 (3)
C1—O4—C4	109.34 (14)	C10—C11—H11	120.1
C5—O5—S1	116.84 (11)	C12—C11—H11	120.1
O1—C1—O4	111.57 (16)	C7—C12—C11	120.5 (3)
O1—C1—C1'	110.53 (16)	C7—C12—H12	119.7
O4—C1—C1'	106.07 (14)	C11—C12—H12	119.7
O1—C1—C2	105.38 (14)	O2—C13—O1	105.86 (14)
O4—C1—C2	106.41 (14)	O2—C13—C15	108.44 (18)
C1'—C1—C2	116.89 (18)	O1—C13—C15	110.09 (19)
O1'—C1'—C1	111.11 (16)	O2—C13—C14	111.1 (2)
O1'—C1'—H1'A	109.4	O1—C13—C14	107.84 (18)
C1—C1'—H1'A	109.4	C15—C13—C14	113.2 (2)
O1'—C1'—H1'B	109.4	C13—C14—H14A	109.5
C1—C1'—H1'B	109.4	C13—C14—H14B	109.5
H1'A—C1'—H1'B	108.0	H14A—C14—H14B	109.5
O2—C2—C3	110.05 (14)	C13—C14—H14C	109.5
O2—C2—C1	103.47 (16)	H14A—C14—H14C	109.5
C3—C2—C1	103.91 (14)	H14B—C14—H14C	109.5
O2—C2—H2	112.9	C13—C15—H15A	109.5
C3—C2—H2	112.9	C13—C15—H15B	109.5
C1—C2—H2	112.9	H15A—C15—H15B	109.5
O3—C3—C4	109.35 (14)	C13—C15—H15C	109.5
O3—C3—C2	109.03 (15)	H15A—C15—H15C	109.5
C4—C3—C2	100.96 (15)	H15B—C15—H15C	109.5
O3—C3—H3	112.3	C21—C16—C17	120.91 (18)
C4—C3—H3	112.3	C21—C16—S1	119.27 (15)
C2—C3—H3	112.3	C17—C16—S1	119.78 (16)
O4—C4—C5	108.83 (15)	C18—C17—C16	119.0 (2)
O4—C4—C3	104.34 (13)	C18—C17—H17	120.5
C5—C4—C3	113.49 (16)	C16—C17—H17	120.5
O4—C4—H4	110.0	C17—C18—C19	121.5 (2)
C5—C4—H4	110.0	C17—C18—H18	119.3
C3—C4—H4	110.0	C19—C18—H18	119.3
O5—C5—C4	106.54 (15)	C18—C19—C20	118.72 (19)
O5—C5—H5A	110.4	C18—C19—C22	120.9 (2)
C4—C5—H5A	110.4	C20—C19—C22	120.3 (2)
O5—C5—H5B	110.4	C19—C20—C21	120.8 (2)
C4—C5—H5B	110.4	C19—C20—H20	119.6
H5A—C5—H5B	108.6	C21—C20—H20	119.6
O1'—C6—C7	109.9 (2)	C16—C21—C20	119.0 (2)
O1'—C6—H6A	109.7	C16—C21—H21	120.5
C7—C6—H6A	109.7	C20—C21—H21	120.5
O1'—C6—H6B	109.7	C19—C22—H22A	109.5
C7—C6—H6B	109.7	C19—C22—H22B	109.5
H6A—C6—H6B	108.2	H22A—C22—H22B	109.5
C12—C7—C8	119.1 (2)	C19—C22—H22C	109.5
C12—C7—C6	121.6 (2)	H22A—C22—H22C	109.5
C8—C7—C6	119.3 (2)	H22B—C22—H22C	109.5
			
O6—S1—O5—C5	178.98 (14)	C1'—O1'—C6—C7	176.95 (18)
O7—S1—O5—C5	48.72 (15)	O1'—C6—C7—C12	−22.5 (3)
C16—S1—O5—C5	−66.49 (15)	O1'—C6—C7—C8	157.6 (2)
C13—O1—C1—O4	103.77 (18)	C12—C7—C8—C9	−0.8 (4)
C13—O1—C1—C1'	−138.45 (17)	C6—C7—C8—C9	179.1 (2)
C13—O1—C1—C2	−11.3 (2)	C7—C8—C9—C10	0.6 (4)
C4—O4—C1—O1	−106.51 (17)	C8—C9—C10—C11	0.0 (4)
C4—O4—C1—C1'	133.06 (17)	C9—C10—C11—C12	−0.4 (4)
C4—O4—C1—C2	7.94 (19)	C8—C7—C12—C11	0.3 (4)
C6—O1'—C1'—C1	−109.0 (2)	C6—C7—C12—C11	−179.6 (2)
O1—C1—C1'—O1'	65.6 (2)	C10—C11—C12—C7	0.3 (4)
O4—C1—C1'—O1'	−173.29 (19)	C2—O2—C13—O1	16.1 (2)
C2—C1—C1'—O1'	−54.9 (3)	C2—O2—C13—C15	134.23 (18)
C13—O2—C2—C3	−132.90 (17)	C2—O2—C13—C14	−100.7 (2)
C13—O2—C2—C1	−22.38 (18)	C1—O1—C13—O2	−2.2 (2)
O1—C1—C2—O2	20.33 (18)	C1—O1—C13—C15	−119.2 (2)
O4—C1—C2—O2	−98.26 (16)	C1—O1—C13—C14	116.9 (2)
C1'—C1—C2—O2	143.52 (16)	O6—S1—C16—C21	−144.04 (16)
O1—C1—C2—C3	135.33 (15)	O7—S1—C16—C21	−12.19 (18)
O4—C1—C2—C3	16.74 (18)	O5—S1—C16—C21	104.34 (15)
C1'—C1—C2—C3	−101.48 (18)	O6—S1—C16—C17	33.81 (17)
O2—C2—C3—O3	−167.94 (15)	O7—S1—C16—C17	165.66 (15)
C1—C2—C3—O3	81.83 (18)	O5—S1—C16—C17	−77.81 (17)
O2—C2—C3—C4	76.98 (18)	C21—C16—C17—C18	2.5 (3)
C1—C2—C3—C4	−33.25 (16)	S1—C16—C17—C18	−175.36 (15)
C1—O4—C4—C5	−151.21 (16)	C16—C17—C18—C19	−0.6 (3)
C1—O4—C4—C3	−29.75 (18)	C17—C18—C19—C20	−2.0 (3)
O3—C3—C4—O4	−76.32 (17)	C17—C18—C19—C22	176.73 (19)
C2—C3—C4—O4	38.51 (16)	C18—C19—C20—C21	2.9 (3)
O3—C3—C4—C5	42.0 (2)	C22—C19—C20—C21	−175.87 (18)
C2—C3—C4—C5	156.83 (15)	C17—C16—C21—C20	−1.6 (3)
S1—O5—C5—C4	165.40 (12)	S1—C16—C21—C20	176.22 (14)
O4—C4—C5—O5	−62.69 (18)	C19—C20—C21—C16	−1.1 (3)
C3—C4—C5—O5	−178.38 (15)		

### Hydrogen-bond geometry (Å, º)

**Table:**

*D*—H···*A*	*D*—H	H···*A*	*D*···*A*	*D*—H···*A*
O3—H3*O*···O4^i^	0.84	1.98	2.812 (2)	174

Symmetry code: (i) *x*, *y*−1, *z*.
